# Effects of Grazing Intensity and Environmental Factors on Species Composition and Diversity in Typical Steppe of Inner Mongolia, China

**DOI:** 10.1371/journal.pone.0052180

**Published:** 2012-12-21

**Authors:** Haiyan Ren, Philipp Schönbach, Hongwei Wan, Martin Gierus, Friedhelm Taube

**Affiliations:** Institute of Crop Science and Plant Breeding-Grass and Forage Science/Organic Agriculture, Christian-Albrechts-University, Kiel, Germany; University of Lausanne, Switzerland

## Abstract

In the present study, we aim to analyze the effect of grazing, precipitation and temperature on plant species dynamics in the typical steppe of Inner Mongolia, P.R. China. By uncoupling biotic and abiotic factors, we provide essential information on the main drivers determining species composition and species diversity. Effects of grazing by sheep were studied in a controlled experiment along a gradient of seven grazing intensities (from ungrazed to very heavily grazed) during six consecutive years (2005–2010). The results show that plant species composition and diversity varied among years but were little affected by grazing intensity, since the experimental years were much dryer than the long term average, the abiotic constraints may have overridden any grazing effect. Among-year differences were predominantly determined by the abiotic factors of precipitation and temperature. Most of the variation in species dynamics and coexistence between C3 and C4 species was explained by seasonal weather conditions, i.e. precipitation and temperature regime during the early-season (March-June) were most important in determining vegetation dynamics. The dominant C3 species *Stipa grandis* was highly competitive in March-June, when the temperature levels were low and rainfall level was high. In contrast, the most common C4 species *Cleistogenes squarrosa* benefited from high early-season temperature levels and low early-season rainfall. However, biomass of *Stipa grandis* was positively correlated with temperature in March, when effective mean temperature ranges from 0 to 5°C and thus promotes vernalization and vegetative sprouting. Our results suggest that, over a six-year term, it is temporal variability in precipitation and temperature rather than grazing that determines vegetation dynamics and species co-existence of grazed steppe ecosystems. Furthermore, our data support that the variability in the biomass of dominant species, rather than diversity, determine ecosystem functioning. The present study provides fundamental knowledge on the complex interaction of grazing – vegetation – climate.

## Introduction

Sustainable grazing management of semi-arid grassland ecosystems requires holistic knowledge about vegetation dynamics and their responses to varying climatic conditions and grazing intensity in order to understand the causes of change in species composition of plant communities. Grazing by livestock is the main anthropogenic disturbance of native grasslands [1] and it plays an important role in determining species composition and plant species diversity [2], [3]. Previous studies have indicated that long-term grazing can affect species diversity of plant communities in grasslands either positively or negatively [4]–[6] but grazing at moderate intensity could promote increased species diversity [7]–[9]. However, in some previous studies the effect of grazing on species diversity may have been overestimated and limited either by the duration of the study or lack of information on grazing intensity and the effects of climatic variability [9]–[11]. Recent studies have shown that environmental moisture, the evolutionary history of grazing, and community productivity also need to be considered when determining the relationships between grazing intensity and species diversity [3], [5].

In semi-arid grassland ecosystems climatic factors are the main drivers that determine plant growth and species dynamics [12]–[14]. Inter-annual variations in precipitation and temperature are reported to be closely correlated to aboveground net primary production and vegetation dynamics (e.g. botanical composition, species diversity) [10], [15]–[17]. However, the vegetation dynamics in semi-arid grasslands have predominantly been analyzed in terms of mean annual precipitation rates and the effect of intra-annual variability in precipitation or temperature remains largely unknown. Little is known about the linkage between temporal variability in precipitation and temperature and the shift in species composition, in particular with regard to the interdependencies between C3 and C4 species. In semi-arid grasslands, initial growth of C4 grasses typically lags behind that of C3 species owing to their requirement for higher temperature in metabolic processes [18]. Thus, C4 species are presumably disadvantaged relative to C3 species in their ability to utilize early spring water resources [19]. In this context, the effects of within-year variation in precipitation and temperature, as well as the interaction between these two abiotic factors, are still not fully understood.

The present study aims to analyze the effect of abiotic (i.e. precipitation and temperature) and biotic factors (i.e. grazing intensity) on the vegetation dynamics in semi-arid grassland of Inner Mongolia, P.R. China. The semi-arid grassland was considered unproductive owing to the relatively low annual precipitation (340 mm) and low aboveground net primary production (under 200 DM g/m^2^) in the context of the MSL (Milchunas, Sala, and Lauenroth [1988]) model [5], [20]. An important aspect was the effect of temporal intra-annual variability of precipitation and temperature and their interactions with the effect of different grazing intensities. The vegetation dynamics were analyzed by the parameters of species diversity and species composition. The relationship between species diversity and ecosystem functioning has been studied for several decades and results have suggested that species diversity may increase, decrease or have no significant effect on primary production [6], [21]–[24]. Furthermore, individual species may respond in different ways to varying environmental factors [25]–[27]. Thus, not only diversity, but also species composition plays an important role in determining ecosystem functioning [28]–[31]. According to the intermediate disturbance hypothesis [7], we assume a positive and negative effect of moderate and heavy grazing, respectively, on species diversity. Furthermore, based on the study of Milchunas and Lauenroth [32], we assume that precipitation has positive effect on species diversity. This may counteract, at least partly, the effect of grazing, due to the current low productivity grassland that has a long evolutionary history. We also hypothesize that increasing temperatures change the proportions of C3 to C4 species according to the different metabolism preferences [18]. In order to comprehensively understand potential shifts in vegetation and underlying processes, it is necessary to uncouple biotic and abiotic determinants of species dynamics in the investigated grazed grassland system. Therefore, a six-year (2005–2010) grazing experiment was conducted to determine: (1) the response of plant species (species composition and diversity) to grazing intensity, varying precipitation and temperature, (2) the effect of temporal within-year variation (annual, seasonal, monthly) in precipitation and temperature on species composition and diversity, and (3) the homeostasis between C3 and C4 species as a function of temporal precipitation and temperature patterns.

## Materials and Methods

### Study Area

The present grazing experiment was conducted during 2005–2010 in a semi-arid grassland of Inner Mongolia, P.R. China, situated near the Inner Mongolia Grassland Research Station of the Chinese Academy of Sciences (IMGERS: 43° 38′N; 116° 42′E, 1200 m a.s.l.). This grassland is classified as typical steppe ecosystem [33]. Based on the long-term meteorological data (1982–2004), the mean annual precipitation of 343 mm and mean annual temperature of 0.7°C characterize a semi-arid, continental climate. This region is characterized by high inter-annual variations in precipitation, which is described by a coefficient of variation (CV) of 22%. Plant growth typically starts between late March and May and lasts until September/October. The typical steppe is dominated by C3 grasses, i.e. *Stipa grandis* P. Smirn. (perennial bunchgrass) and *Leymus chinensis* Trin. Tzvel. (a perennial rhizomatous grass) which together account for approximately 60–80% of the total community aboveground biomass in the experimental area. The most common C4 species is *Cleistogenes squarrosa* (Trin.) Keng, which accounts for 3–9% of the community aboveground biomass (Table 1).

**Table 1:** Effect of grazing intensity (GI) and year on aboveground biomass (g DM m^−2^) of species and diversity indices (richness, diversity and evenness).

GI	*L.ch*	*S.gr*	*C.sq*	Richness	Diversity	Evenness
0	33.5	55.0	6.1	11.5	1.4	0.6
1	71.0	33.2	3.1	13.1	1.3	0.5
2	42.5	56.7	6.1	10.8	1.3	0.5
3	21.5	62.6	5.7	9.5	1.2	0.5
4	36.4	38.3	4.5	12.4	1.4	0.6
5	19.8	47.2	5.6	9.9	1.3	0.6
6	20.8	37.5	3.0	11.3	1.3	0.6
YEAR						
2005	45.4	26.4	9.8	14.1	1.6	0.6
2006	30.2	26.2	7.8	13.0	1.5	0.6
2007	40.4	38.1	2.4	10.4	1.2	0.5
2008	42.0	48.8	4.3	12.4	1.2	0.5
2009	29.7	53.1	3.1	8.1	1.2	0.6
2010	40.0	73.5	1.7	9.3	1.1	0.5
*F*-values statistics for the test of particular analysis						
GI	3.6*	1.9 ns	0.7 ns	0.9 ns	1.1 ns	0.4 ns
YEAR	1.9 ns	8.3***	12.4***	14.7***	12.3***	3.9**
BLOCK	15.4**	1.0 ns	0.2 ns	2.1 ns	0.8 ns	0.0 ns
GI×YEAR	0.6 ns	1.7 ns	1.5 ns	1.1 ns	2.0 ns	1.7 ns

The abbreviations are: *L.ch* (*Leymus chinensis*), *S.gr* (*Stipa grandis*), *C.sq* (*Cleistogenes squarrosa*).

(*: 0.01<*P*<0.05, **: 0.001<*P*<0.01, ***: *P*<0.001, and ns means not significant, *P*>0.05).

### Experimental Design and SAMPLING

An area of 150 ha of natural grassland was fenced and split into 2-ha sized paddocks. Seven grazing intensities (GI) (no grazing (GI0), very light (GI1), light (GI2), light-moderate (GI3), moderate (GI4), heavy (GI5) and very heavy (GI6) grazing) were randomly distributed to 2-ha sized paddocks. A gradient of herbage allowances was used to modulate the grazing intensities. The seven GI corresponded to different herbage allowance classes ranges from <1.5, 1.5–3, 3–4.5, 4.5–6, 6–12 to >12 kg dry matter (DM) kg^−1^ live weight (LW) of sheep. Due to the spatial heterogeneities and the varied available herbage on offer between the plots, the numbers of sheep were adjusted monthly to achieve herbage allowance target ranges [12], [16] and thus to keep the grazing pressure of each grazing intensity constant by using the put-and-take stocking method [34]. Further details on herbage allowances, stocking rates and the grazing intensity classification are given in Schönbach *et al.* [20]. GI treatments were replicated on two blocks according to the topographic positions: one was on a level area and the other on slopes. The area has a long history of being managed at moderate to heavy grazing intensity levels, but livestock had been excluded from this area two years prior to the start of the experiment in 2005. Sheep grazing lasted from June to September for 98, 90, 93, 94, 94 and 95 days in 2005, 2006, 2007, 2008, 2009 and 2010, respectively. The duration of grazing was in accordance with the common local grazing season.

On each treatment plot, three representative sampling areas were chosen before the grazing period started in June. In each sampling area, exclosure cages (2 × 3 m) were set up and moved at monthly intervals, subsequent to each sampling, to locations that had been grazed previously. The aboveground community biomass was sampled monthly (June, July, August and September) inside and outside of the exclosures, with sampling taken inside a 0.25 m × 2 m rectangular frame ( = 3 × 0.5 m^2^ frames). Aboveground biomass sampling of each species was done once a year at peak biomass in July inside the exclosures for determination of botanical composition (i.e., species biomass sampled was not subjected to current-year grazing). Plant species were recorded as present/absent for the purpose of assessing species richness (R) inside the rectangular frames, then clipped to 1 cm stubble-height, separated by species, and taken to the laboratory for quantitative dry matter determination after drying at 60°C for 48 h.

‘Species composition’ comprises aboveground biomass of the six most common grass species sampled (*L. chinensis*, *S. grandis*, *C. squarrosa*, *Carex korshinskyi*, *Agropyron cristatum*, and *Achnatherum sibiricum*). Other plant species that comprised less than 1% of the biomass were allocated to the fraction termed “remaining species”. Species diversity was characterized by three different parameters. The number of plant species and plant biomass density of each species in each plot was counted to calculate (i) the ‘species richness’ (R), (ii) the ‘Shannon–Wiener diversity index’ (D) (see equation 1), (iii) and the ‘species evenness’ (E) (see equation 2)
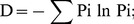
(1)

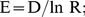
(2)


Where Pi is the weighted average proportional biomass density of the *i* species.

### Meteorological Data

In order to determine the relationship of vegetation dynamics with precipitation and temperature, three different scales of temporal variation were investigated: annual, seasonal, and monthly. Seasonal partitioning comprises three periods: early-season (March-June), late-season (July-October) and out-of-season (November-February) (Table 2). In order to increase the level of detail, early-season precipitation and temperature were further divided into four sub-periods, i.e. March, April, May and June (Table 3). Thus, precipitation sums and temperature means during certain periods were used to analyze their relationship with vegetation dynamics.

**Table 2:** Precipitation (PPT in mm) and mean temperature (MT in °C d^−1^) in early season (March-June), late season (June-October) and out of season (October-March) in six experimental years.

	Early-season		Late-season		Out-of-season	
Year	MT	PPT	MT	PPT	MT	PPT
2005	8.8	68.7	1.9	230	−15.9	13.6
2006	8.4	130	0.6	79.0	−16.9	18.7
2007	9.8	131	1.3	154	−15.4	31.5
2008	9.6	146	0.7	86.8	−16.6	17.5
2009	8.6	111	1.6	196	−14.5	16.7
2010	6.9	116	1.1	131	−16.9	2.1
Mean	8.7	117	1.2	146	8.7	16.7

Early season: March to June; Late season: July to October; Out of season: November to February.

**Figure 1:**
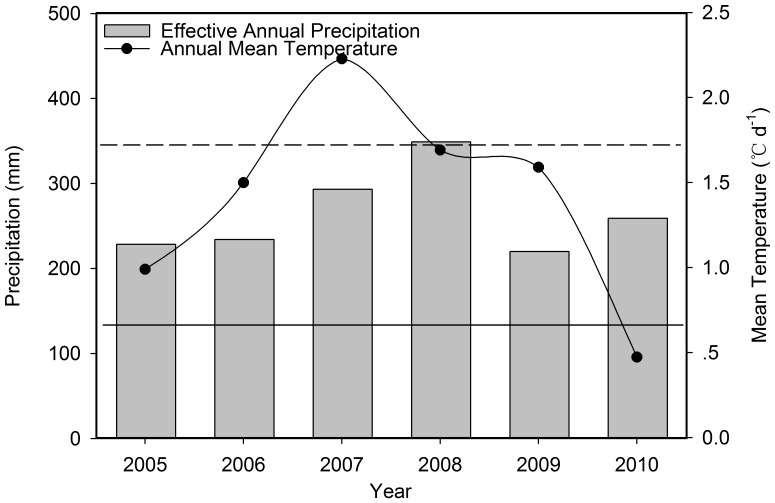
Effective annual precipitation rates (left y-axis) and annual mean temperature (right y-axis) from 2005–2010. The horizontal dashed line denotes the 20-year (1983–2004) mean effective annual precipitation of 343 mm, and the horizontal solid line denotes the mean annual temperature of 0.7°c over the same 20–year period.

The annual effect of precipitation was determined by using the effective annual precipitation (previous-year September to current-year September) instead of using calendar annual (January to December) sums (Figure 1). This was because the previous-year precipitation during winter was likely to have affected species composition in the current growing season [36]. On a monthly basis, the mean daily temperatures above 0°C were used to calculate effective mean temperature for March, April, May and June; whereas for seasonal and annual periods all temperature values were included.

Apart from the above-mentioned mean temperature, further temperature indices (e.g. active accumulated temperature) were tested by multiple stepwise regression analysis (see statistics section) regarding their effect on vegetation dynamics. However, in this study we focus only on the indices that explained most of the variances in vegetation dynamics, i.e. precipitation sums during specified periods, effective annual precipitation (previous-year September to current-year September), mean temperature during specified periods and effective mean temperature in individual months.

The decision to use effective mean temperature rather than actual mean temperature on a monthly basis was made, because mean temperatures in March and April in this region are below 0°C, and plant species will not grow under this temperature.

During the six experimental years (2005–2010), effective annual precipitation varied from a minimum of 219 mm (2009) to a maximum of 345 mm (2008) and annual mean temperature ranged from a minimum of 0.5°C (2010) to a maximum of 2.2°C (2007) (Figure 1). In comparison with the long-term average (1982–2004) of 343 mm, the effective annual precipitation rates during the experimental period (2005–2010) were relatively low (Figure 1). The opposite is true for annual mean temperature during the experimental period, which was above the long-term average of 0.7°C for most years (Figure 1). During the 6 experimental years, the mean annual precipitation and temperature are 264 mm and 1.4°C, respectively, and the CV is 19%. Temporal patterns of precipitation and temperature were observed from 2005 to 2010 (Table 2 and 3). During the early-season, monthly precipitation and effective mean temperature (above 0°C mean daily temperature) varied strongly within and among years (Table 3).

**Table 3:** Monthly precipitation (PPT in mm) and effective mean temperature (EMT in °C d^−1^) during the early season (i.e. March, April, May, June) in six experimental years.

	March		April		May		June	
Year	EMT	PPT	EMT	PPT	EMT	PPT	EMT	PPT
2005	1.7	1.7	6.5	9.2	10.4	3.8	17.8	54.0
2006	2.4	2.5	5.8	4.1	11.7	62.7	16.4	60.5
2007	3.7	16.4	4.3	16.8	11.4	32.3	20.4	65.4
2008	3.0	15	7.2	25.2	9.7	32.3	17.2	73.1
2009	3.9	1.3	5.5	51.7	12.8	10.6	14.9	47.2
2010	3.1	3.4	3.3	40.7	10.7	50.4	17.8	21.5
Mean	2.9	6.7	5.4	24.6	11.1	32.0	17.4	53.6

Effective mean temperature: above 0°C mean daily temperature in the respective period of time.

### Statistics

The experimental design was a randomized complete block design with two replicates, i.e. one on a level area and one on a sloping area. All statistical analyses were carried out in SAS, Version 9.1 (SAS Institute Inc., Cary, NC, USA). Simple linear regression was used to test the correlation between the biomass of the target species and diversity parameters. The effects of grazing intensity, year and their interactions on species composition and species diversity were analyzed by using the residuals of diversity-species biomass linear regression in the Repeated Measures ANOVA - mixed model. The statistical model included grazing intensity, block, year and their interactions as fixed effects. The factor year was used as repeated effect and grazing intensity × block as random effect. ‘Block’ was a fixed effect because treatments were replicated on a level area as well as on slopes, and then considered as block level and block slope. Thus block was not randomly chosen but explicitly assigned to two out of several typical areas with different environmental conditions. The level of significance was *P*<0.05. Owing to the limitation of the ANOVA mixed model to deal with quantitative determining variables, the effects of seasonal variations in precipitation and temperature were tested by Pearson correlation and multiple regressions. The Pearson correlation analysis was used to examine the associations of species composition and diversity parameters (richness, diversity index and evenness) with the variation of both the precipitation rates and mean temperature at annual and seasonal level. The multiple regression model with stepwise selection was additionally applied to in order to provide quantitative information on the correlations and importance of precipitation and temperature on species biomass and diversity parameters. Variables of precipitation and temperature during certain periods of time were selected, and the variable that improved the model the most (determined by higher R-square and significance at the 0.05 level) was maintained by using stepwise selection model comparison criterion. The stepwise-building used forward selection and the variables already built in could not be thrown out at a later stage. The coefficient of determination R-square was tested with a two-sided test for significance of the stepwise regression model (*P*<0.05). By comparing the model coefficients of determination, the optimum model accounting for maximum variation was chosen.

## Results

### The Effects of Grazing Intensity and Years

The aboveground biomass of dominant species *L. chinensis* significantly decreased with increased grazing intensity, while other investigated species and species diversity parameters (richness, Shannon-Wiener diversity index and evenness) were not significantly affected by grazing intensity (Table 1). However, species composition and diversity varied significantly among experimental years over all grazing intensities. The biomass of the dominant C3 grass *S. grandis* was two-fold higher in the final year 2010 compared with the first experimental year of 2005, whereas the biomass of the C4 species *C. squarrosa* and C3 species *A. sibiricum* decreased by 83% and 60% respectively. The species grouped as ‘remaining species’ contributed the highest amount of biomass in the initial year 2005 and reached the lowest value in 2009. In comparison to 2005, species richness, diversity and evenness in 2010 decreased by 34, 31 and 16%, respectively. No significant interactions between year and grazing intensity on species composition and diversity were detected.

### The Effects of Precipitation and Temperature

Pearson correlation analysis showed that calendar year-based annual precipitation and mean temperature had no significant effect on almost all measures of species biomass. The exception was *C. squarrosa* which was negatively correlated with annual precipitation. There was also no significant relationship between the variations of both effective annual precipitation and mean temperature and diversity (except richness) (Table 4). On a seasonal scale, most of species biomass and all diversity parameters were correlated with early-season (March to June) precipitation and mean temperature, whereas precipitation and temperature conditions during late-season (July to October) and out-of-season (November to February) had only little effect on species dynamics (Table 4).

**Table 4:** Correlation coefficients (*n = *84) for relationship between aboveground biomass of each species and diversity indices (richness, diversity and evenness) with precipitation (PPT in mm) and mean temperature (MT in °C d^−1^).

Parameters	Early season (Spring)		Late season (Summer)		Out of season (Winter)		Annual	
	PPT	MT	PPT	MT	PPT	MT	PPT	MT
*L.ch*	−0.1 ns	0.0 ns	0.1 ns	−0.1 ns	−0.0 ns	−0.1 ns	0.1 ns	0.1 ns
*S.gr*	0.3*	−0.3**	−0.1 ns	−0.2 ns	−0.2*	−0.0 ns	0.2 ns	0.1 ns
*C. sq*	−0.3**	0.2*	0.1 ns	0.0 ns	0.0 ns	−0.1 ns	−0.2*	−0.1 ns
Richness	−0.3**	0.2*	−0.1 ns	0.1 ns	0.1 ns	−0.1 ns	−0.1 ns	0.2*
Diversity	−0.3**	0.2*	0.1 ns	0.1 ns	0.1 ns	−0.1 ns	−0.1 ns	−0.2 ns
Evenness	−0.3*	−0.0 ns	0.2 ns	−0.0 ns	0.0 ns	0.1 ns	−0.1 ns	−0.2 ns

Early-season: March to June; Late-season: July to October; Out-of-season: November to February.

(*: 0.01<*P*<0.05, **: 0.001<*P*<0.01, ***: *P*<0.001, and ns means not significant, *P*>0.05).

For abbreviations of species names see Table 3.

Multiple regression analysis indicated that early-season precipitation and early-season mean temperature strongly correlated with *S. grandis*, *C. squarrosa* and *A. sibiricum* biomass, ‘remaining species’ biomass and diversity parameters (Table 5). The coefficients of determination from the multiple stepwise regression analysis showed that early-season precipitation and early-season mean temperature accounted for most of the variations in species composition and diversity. Precipitation and mean temperature during the early-season together explained 12–42% of the variation in species biomass and 11–40% of the variation in diversity (Table 5). The dominant C3 species *S. grandis* responded positively to increasing early-season precipitation and negatively to early-season mean temperature, whereas the opposite was true for all other species and for all diversity parameters (Table 5).

**Table 5:** Partial R-square coefficients of determination (*n = *84) associated species diversity and species composition with precipitation (PPT in mm) and mean temperature (MT in °C d^−1^) in the early-season (March - June) for stepwise variance regression analysis.

Parameters		*L.ch*	*S.gr*	*C.sq*	Richness		Diversity		Evenness
Partial R^2^ coefficients of determination									
Precipitation (mm)		0.0 ns	0.1**	0.3***	0.2***		0.3***		0.1**
Temperature (°Cd^−1^)		0.0 ns	0.2***	0.1**	0.1**		0.1**		0.0 ns
	**Model Equation**					**Adjusted R^2^**		***P***	
*S. grandis*	Y*_S.gr_* = 0.186PPT - 0.234MT +103.686					R^2^ = 0.3		*P*<0.0001	
*C. squarrosa*	Y*_C.sq_* = − 0.036PPT +0.024MT +15.726					R^2^ = 0.4		*P*<0.0001	
Richness	Y_Richness_ = − 0.017PPT +0.017MT +9.755					R^2^ = 0.3		*P*<0.0001	
Diversity	Y_Diversity_ = − 0.002PPT +0.001MT +1.763					R^2^ = 0.4		*P*<0.0001	
Evenness	Y_Evenness_ = − 0.001PPT +0.714					R^2^ = 0.1		*P*<0.01	

For abbreviations of species names see Table 3.

(*: 0.01<*P*<0.05, **: 0.001<*P*<0.01, ***: *P*<0.001, and ns means not significant, *P*>0.05).

**Figure 2:**
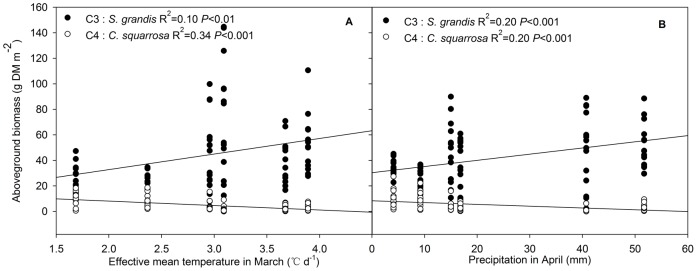
Relationship between aboveground biomass (g DM m^−2^) of the C3 species *S. grandis* and C4 species *C. squarrosa* and effective mean temperature (°C d^−1^) in March (A) and precipitation rates (mm) in April (B) (*n* = 84, i.e. 14 observations per year).

In order to increase the level of detail, early-season precipitation and temperature were further subdivided into monthly periods of March, April, May and June. Multiple stepwise regression analysis revealed a strong linkage of monthly precipitation and effective mean daily temperature with the biomass of *S. grandis* and *C. squarrosa* and diversity (Table 6). In March, the variability in the biomass of the C3 species *S. grandis*, the C4 species *C. squarrosa* and in diversity were correlated only with effective mean daily temperature but not with precipitation. Meanwhile, inverse responses were found as the biomass of the C3 species *S. grandis* increased while that of the C4 species *C. squarrosa* and any of the diversity parameters decreased with increasing effective mean daily temperature in March (Fig. 2A, Table 6). In April, it was precipitation rather than temperature that determined vegetation dynamics, i.e. C3 species *S. grandis* biomass increased while C4 species *C. squarrosa* biomass and any of the diversity parameters decreased with increasing precipitation (Fig. 2B, Table 6). By combining precipitation and effective mean daily temperature of March and April, 30% of the variance in the biomass of the C3 species *S. grandis*, 59% of that of the C4 species *C. squarrosa*, and 60 and 54% of the variance of richness and diversity, respectively, can be explained. In May, no significant correlation relationship was found for any parameter. In June, only the aboveground biomass of the C3 species *S. grandis*, richness and diversity were correlated with precipitation, and the coefficients of determination were low (Table 6).

**Table 6:** Adjusted R-Square coefficients of determination (*n = *84) associated C3 vs. C4 species (*S. grandis* vs. *C. squarrosa*) and diversity with precipitation (PPT in mm) and effective mean temperature (EMT in °C d^−1^) for multiple stepwise variance regression analysis.

	March			April			June		
Parameters		R^2^	*P*		R^2^	*P*		R^2^	*P*
*S. grandis*	Y = 12.174EMT+8.501	0.1	0.0038	Y = 0.76PPT+26.917	0.2	<0.0001	Y = 0.678PPT+80.653	0.2	0.0003
*C. squarrosa*	Y = −3.513EMT+15.190	0.3	<0.0001	Y = −0.085PPT+0.914EMT+1.829	0.3	<0.0001	NS	NS	>0.05
Richness	Y = −2.487EMT+18.536	0.3	<0.0001	Y = −0.112PPT+13.778	0.3	<0.0001	Y = 0.066PPT+7.679	0.1	0.0036
Diversity	Y = −0.217EMT+1.936	0.3	<0.0001	Y = −0.008PPT+1.488	0.2	<0.0001	Y = 0.004PPT+1.067	0.1	0.0287
Evenness	Y = −0.035EMT+0.670	0.1	0.0088	NS	NS	>0.05	NS	NS	>0.05

“NS” means no variable met the 0.05 significance level for entry into the model.

Effective mean temperature: above 0°C mean daily temperature in the respective period of time.

## Discussion

### The Effects of Grazing Intensity and Years

An aim of this study was to investigate whether grazing intensity has a strong effect on species composition and diversity in the Mongolian steppe. Our results demonstrate that grazing intensity has relatively little effect on species composition; only the aboveground biomass of *L. chinensis* differed among grazing intensities, and no diminishing effect on species diversity was observed. Also, grazing intensity had no effect on cumulative biomass of species and diversity over six consecutive years of grazing. The results obtained, therefore, do not support the intermediate disturbance hypothesis, which assumes that moderate grazing intensity increases species diversity and that high grazing intensity leads to a shift in the proportion of dominant and opportunistic species [7], [8], [32]. It has been suggested that long-term grazing induces a significant shift in the aboveground biomass composition of Inner Mongolian grasslands, from perennial C3 grasses, such as *S. grandis* or *L. chinensis*, to C4 grass species such as *C. squarrosa* (e.g. [18], [36]). Thus, the time scale over which the grazing effects are analyzed plays an important role in determining whether or not grazing affects vegetation dynamics. According to the findings of many previous studies, and as suggested by the above-cited references [7], [8], [18], [32], [36], shifts in the botanical composition and species diversity are likely to occur under long-term grazing, i.e., during periods of at least ten years duration. Long-term experiments are indeed very valuable but also very rare. Transect studies provide an alternative to highlight grazing effects. Although these studies have to consider the unfairly heterogeneous areas, such as different vegetation unit, steppe type, species richness and soil parameters, they allow assessments covering historical old gradients of grazing activity, as some work already done in the same region, e.g. [9], [37], [38]. However, our findings suggest that vegetation dynamics are much less influenced by grazing in the medium-term, as most of the investigated species and all of the analyzed diversity indices remained unaffected by the factor of grazing intensity. The long history of grazing in the study region may have led to the development of a steppe vegetation community that was adapted to and tolerant of grazing [3]. It has also been considered likely that selective grazing by sheep and the competitive balance among species (e.g. various grazing resilience abilities and regrowth rates of species) counteract the effects of grazing in the medium-term. Furthermore, during the experimental period, annual precipitation patterns in the study region were much lower than the long-term average. Most of the species present, and their composition, may have been well adapted to more average conditions. Therefore, the current grazing system was perhaps untypical for the conditions with abiotic constraints, including droughts. Conversely, the biotic constraints of grazing may become more effective under more average conditions. The long evolutionary history of grazing and low habitat productivity as a result of low environmental moisture on this study site have contributed to the grazing intensity-diversity relationship fitting the MSL (Milchunas, Sala, and Lauenroth [1988]) original equilibrium curves. According to our results, the aboveground biomass of the plant species investigated here respond differently to the effects of grazing. The pronounced decrease in the aboveground biomass of *L. chinensis* with increased grazing intensity was presumably a result, at least in part, of its relatively high forage quality [39]–[41]. In consequence, sheep preferentially graze *L. chinensis* so that the aboveground standing biomass of this species was significantly reduced with increased grazing intensity. In contrast, *S. grandis* is characterized by a relatively low nutritive forage value owing to high concentrations of fibre and lignin and thus has low digestibility [39]. Therefore, *S. grandis* is less-preferentially grazed by sheep compared with *L. chinensis* [42], [43]. However, it should be taken into account that current-year species sampling was done in July inside exclosures, and therefore any grazing-related effects on species would have been determined by the previous year’s grazing, rather than grazing in the current year. Furthermore, not only grazing preference will have determined the grazing effect on the aboveground biomass of plant species, but also the different strategies of plants to compensate for herbage removal under grazing. For example, *C. squarrosa* is characterized by a relatively high nutritive forage value [39], but nevertheless grazing intensity had no effect on aboveground biomass of this major C4 species. We therefore assume that the strong regrowth ability (grazing tolerance) of *C. squarrosa* [39] compensated for the removal of its biomass under grazing.

In the present study, vegetation dynamics were predominantly determined by inter-annual differences and to a lesser extent by grazing intensity. Owing to the weak correlation between the investigated vegetation parameters and annual precipitation (data not shown), the decrease of species diversity over time may not be attributed to the cumulative effects of persistent droughts. Even though the precipitation increased from 2005 to an average level in 2008, species diversity did not show increase with higher precipitation. This result did not support our assumption that precipitation leads to species diversity increasing. Thus, precipitation and temperature factors and their inter-annual variability have to be taken into account when analyzing the complex competition and colonization processes of grazed steppe vegetation [10], [32], [44]. In our study, the vegetation was subjected to six years of grazing at different intensity levels. Therefore it should be considered as a starting point during the long-term dynamic process of vegetation formation in grazed grasslands. The pronounced variability in species composition and diversity among years supports the hypothesis that inter-annual variability in precipitation and temperature predominantly determines vegetation responses to grazing [10]. Therefore, the effect of year is discussed in the following sub-section in the context of precipitation and temperature.

### The Effects of Precipitation and Temperature

Our results highlight the importance of temporal distribution patterns of precipitation and temperature on vegetation dynamics. Most of the variation in species composition and diversity was determined by precipitation rates and temperatures in the early season (March to June), whereas only a small proportion of the variance in the investigated vegetation parameters could be explained by precipitation rates and mean temperature on an annualized scale or in late-season (July to October) or out-of-season (November to February) period. In terms of the responses of species composition (slowly and long-term) and plant productivity (fast and short-term) to environmental influences, previous studies also reported that the temporal distribution, rather than the annual sum of precipitation, determines aboveground net primary productivity of semi-arid grasslands [45], [46]. Thus, seasonal distribution of precipitation and temperature presented in the following paragraphs are considered when analyzing vegetation dynamics in steppe ecosystems. Stepwise regression analysis confirmed that vegetation dynamics are strongly affected by precipitation and mean temperature in the early season (March to June). In this period, most species initiate their growth processes and thus they are highly sensitive to variations in precipitation and temperature. According to our results, a combination of those two factors (precipitation and temperature) explains most of the variation in species diversity (R^2^ = 0.32–0.40) and species composition (R^2^ = 0.12–0.42). However, species varied in their response to precipitation and temperature. For example, the early-season mean temperature (R^2^ = 0.18) was the main factor determining the aboveground biomass of the dominant C3 species *S. grandis*, with a slightly greater effect than early-season precipitation (R^2^ = 0.13) (Table 4). In contrast, aboveground biomass of *C. squarrosa* was predominantly determined by early-season precipitation (R^2^ = 0.34) and to a lesser extent by early season-temperature (R^2^ = 0.09) (Table 4). The inverse impact of temperature and precipitation on plant species in the Inner Mongolian steppe is presumably a result of their different sensitivity to temporal fluctuations of precipitation or temperature variability and thereby diverse behavior and strategies to adapt to the habitats (e.g. high photosynthetic capacity and high resource-use efficiency of C4 species in the dry environment and high freezing-resistance of C3 species in the frozen environment). In order to predict and explain shifts in plant species composition, both precipitation rates and temperature sums during the early season need to be taken into account.

The inverse responses of the C3 species *S. grandis* and C4 species *C. squarrosa* to early-season precipitation and temperature support the temporal niche differentiation theory, which claims that the competitive balances of C3 and C4 species are related to temporal variances of climatic factors [47]. Considering that C3 and C4 species have different phenological (reproductive) development as well as different competitive strategies (e.g. trade-off between fast growth and nutrient storage) within a given growing stage, this would change their final survival [18], [48], the effects of monthly distribution of precipitation and effective mean daily temperature were tested further. The results show that March and April were two very important months for C3 and C4 species and for species diversity for the upcoming growing period. In March, the main driver was the effective mean temperature. In April, the main driver was precipitation. The shift from temperature to precipitation between March and April may be attributed to the main determining factor changing in response to the different growth stage of plant species, as reported by Lundholm and Larson [49]. Taking the effects in these two months together, 59% of the aboveground biomass variance of C4 species *C. squarrosa* and 54–60% of the variance in diversity can be explained.

From March to June, the responses of species to precipitation and effective mean temperature vary. In the early spring phenophases during March, the stimulating of propagation in *S. grandis* occurred. This included seeds germinating and vegetative sprouting [50], [51]. The seeds of *S. grandis* were presumably stimulated by low temperature for vernalization and breaking dormancy [50], [52]–[54]. The vegetative sprouting of *S. grandis* was also presumably stimulated by effective mean temperature for a period of warm stratification after the cooler winter temperatures [50], which resulted in the spring phenophases moved earlier and promoted lengthening of its growing season. Thus the competitor C3 species *S. grandis* started its growth in March one month earlier than C4 species *C. squarrosa*, when the effective mean daily temperatures in March (<4.0°C) are suboptimal for growth of C4 plants but are optimal for C3 species vernalization and sprouting. The C4 species *C. squarrosa* required higher temperature to activate tillers and initiate new-season growth, and higher temperature for metabolism than C3 species like *S. grandis*. For this reason *S. grandis* is able to exhibit a superior metabolic performance at low temperatures [18], [55]. In April, the precipitation amount increased and the C3 species *S. grandis* could make full use of rainfall for aboveground biomass growth, while the C4 species *C. squarrosa* was unable to grow, owing to its delayed spring initial growth and shallow rooting depth of only 20 cm [56]. In May, in order to provide enough energy for later flowering and fruiting, species gradually started to accumulate carbohydrate in their root system, so that they need less precipitation and temperature for aboveground biomass growth [35]. This may explain why no significant correlation was found between precipitation and temperature factors in May and species aboveground biomass. In June, the C3 species *S. grandis* turns moves into its flowering and fruiting period and thus needs water to continue growing, but the C4 species *C. squarrosa* remains in the root carbohydrates accumulating period. This may explain the positive response of C3 species and the lack of response of C4 species to precipitation in June. Information on the temporal dynamics of species composition regarding C3 and C4 species is required in order to formulate carrying capacities and predict future change of C3 and C4 species with specific temporal distribution of precipitation and temperature variation, as well as to develop appropriate management practices.

Species diversity parameters were observed to have the same trends as the C4 species *C. squarrosa* to either early-season or monthly distributions of temperature and precipitation. The results suggest that not just C4 species, but other subdominant species and rare species are contributing to the species diversity, have similar mechanisms to stay competitive against the trade-off with dominant species, according to temporal distribution of environmental factors. The dominant species *S. grandis* is generally known for its root biomass being several times larger than the above-ground biomass [18]. It may prove to be highly competitive to maintain its population in the community because it occupies resource space for long periods, and therefore captures the chance of other species for contributing to early season production. Under persistent limited resource availability, the semi-arid grassland may be evenly distributed by dominant species in the long term, as species richness and evenness decreased over time. The similar responses of C4 species’ biomass and diversity parameters and inverse responses of C3 species *S. grandis*’ biomass to environmental factor effects are consistent with the result confirmed by our data that there is no significant correlation between diversity parameters and community biomass (R^2^_richness_ = 0.06; R^2^_ diversity_ = 0.03; R^2^_ evenness_ = 0.10) (detailed data not shown). From another point of view, since controversial arguments about the relationship between species diversity and community productivity or ecosystem stability are presented, the mass ratio hypothesis, which holds that ecosystem functioning and stability is largely controlled by dominant species rather than diversity in the medium-term, supports our results [57]. Since dominant species could also control the effect of diversity on community stability, our finding that the variances of the most productive species *S. grandis* influenced species dynamic more than species diversity is considered to be evidence for this.

### Conclusions

We find that temporal distribution of precipitation and temperature, in particular during the early-season (from March to June), determined the variation in vegetation dynamics much more strongly than annual rates of precipitation or temperature. In the medium term (6 years), the effects of early-season precipitation and temperature on the trade-off among species were more important than the effect of grazing. However, grazing decreased the aboveground biomass of those species that are more preferentially grazed by sheep, such as *L. chinensis*. Therefore, based on these findings, the effect of grazing in a grassland system cannot be analyzed without considering the characteristic traits of species and also the precipitation and temperature variation, especially in the medium term. Our findings demonstrate that the temporal fluctuation of precipitation and temperature, especially in early season, seems to provide more robust and comprehensive predictions on vegetation dynamics in semi-arid typical steppe, and helps to understand species coexistence and grassland management in response future extreme precipitation and drought events.
